# A Rare Presentation of Metastatic Breast Cancer Manifesting As Diffuse Nodular Skin Lesions

**DOI:** 10.7759/cureus.49633

**Published:** 2023-11-29

**Authors:** Henrik Ghantarchyan, Nia Abbas, Azaria Lewis, Christopher Chamanadjian, Raoufi Kambiz

**Affiliations:** 1 Internal Medicine, Arrowhead Regional Medical Center, Colton, USA

**Keywords:** skin biopsy, pr positive, er positive, skin nodules, metastatic breast cancer

## Abstract

We present a 63-year-old African American female with a prior medical history of gastroesophageal reflux disease (GERD) and uterine fibroids whose primary concern was vaginal bleeding. She had no prior medical care established, and the last mammogram was conducted 10 years prior with normal results. She has had multiple ED visits for symptoms of reflux and vaginal bleeding and has been discharged with a primary care follow-up referral each time. On physical exam, there was evidence of nodular skin lesions, tightening of the skin on her face, neck, and back, as well as nodular skin lesions on her neck, back, chest, and abdomen, notably progressing in number, not in size, in a caudal fashion. Further exam findings included telangiectasias predominantly on her right hand. On initial laboratory studies, she was hypercalcemic with an elevated calcium level of 13 mg/dL. Initial imaging included a CT scan of her chest, abdomen, and pelvis, which revealed pulmonary embolism and uterine fibroids, with the largest measuring 5.9 x 4.3 x 5.3 cm, as well as bilateral breast masses noted to be a BI-RADS 3 on ultrasound. A skin biopsy completed early on in the hospitalization revealed metastatic breast cancer, specifically high-grade, poorly differentiated infiltrating mammary carcinoma of the lobular type. Similarly, a right breast mass biopsy illustrated resemblant findings, specifically invasive mammary carcinoma with mixed ductal and lobular features. She was ultimately treated with ribociclib and fulvestrant (KR1) and discharged from the hospital with oncology and primary care follow-up.

## Introduction

The incidence of cutaneous metastases is about 10% of oncologic conditions, with the most common being melanomas, squamous cell carcinoma of the head and neck, and breast cancer [1]. Typically, cutaneous metastases appear two to three years after the initial diagnosis [2]. However, cutaneous metastases can present as the primary manifestation of previously undiagnosed malignancy or recurrent cancer [3].

Cutaneous metastases frequently present as multiple or single nodules, less frequently as plaques, and least frequently as ulcers [4]. We present a case of a 63-year-old female patient suffering from diffuse nodular cutaneous metastases located on her anterior and posterior neck, chest, and bilaterally on her breasts. This initially presented as a rash on her anterior neck when she sought medical attention for worsening abdominal pain and gastroesophageal reflux disease (GERD). As she was discharged from the ED, a dermatology referral request for a punch biopsy of her anterior neck rash was given. The patient shared the ED discharge paperwork with her primary care provider (PCP) and requested a referral to dermatology; however, her PCP believed a dermatological referral was not necessary. Ultimately, the lack of recognition and diagnostic workup of the cutaneous metastases delayed diagnosis and potential treatment. For this reason, we drafted this case report in hopes of increasing clinical awareness of breast cancer metastasis to the skin as an initial sign of breast cancer to be investigated both early and thoroughly.

## Case presentation

**Figure 1:**
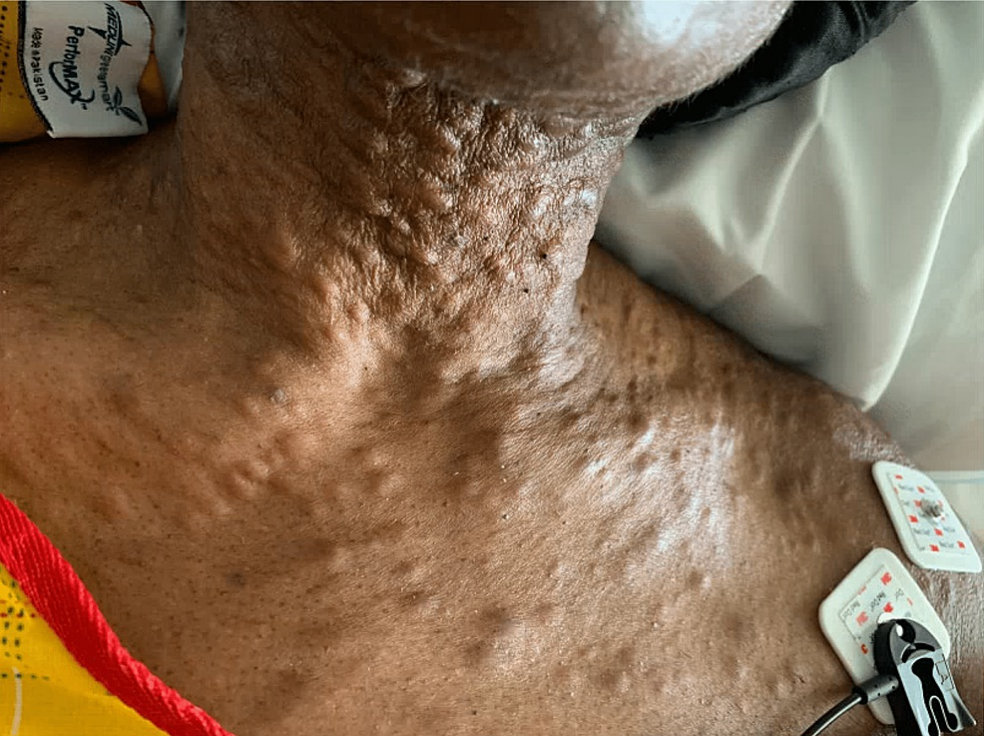
Nodular skin lesions

We present a case of a 63-year-old African American female with newly diagnosed GERD who presented with a four-month history of abdominal pain, a two-month history of significant vaginal bleeding, nausea, vomiting, fatigue, and a 20-pound unintentional weight loss. At the time of admission, the patient was using 8-10 perineal pads per day for increased post-menopausal bleeding. The patient also noted raised skin lesions that initially developed on the anterior neck and had progressed to cover her posterior neck, chest, back, and abdomen (Figure 1). She had been to the ED multiple times for abdominal pain and dysphagia and was subsequently diagnosed with fibroids and GERD. Prior to the ED visits, the patient had not seen a PCP for 10 years. At the previous ED visits, a CT scan and ultrasound of the uterus showed multiple fibroids, with the largest measuring 5.9 x 4.3 x 5.3 cm and a left ovarian cyst. The patient denied any family history of cancer. Objectively, the patient seemed ill with obvious signs of temporal wasting. She complained of significant, diffuse abdominal tenderness. Bilateral breast masses were palpated in addition to adenopathy, mainly in the cervical and axillary areas. A skin exam revealed diffusely raised nodules on the anterior and posterior neck, chest, back, and abdomen. The nodular skin caused a thickening of her skin, which limited movement in her neck and jaw to the point that she had difficulty opening her mouth to speak or eat.

Laboratory investigations were within normal limits except for marked hypercalcemia at 13 mg/dL. The patient received zoledronic acid and IV fluid resuscitation for the treatment of hypercalcemia. Parathyroid and parathyroid-related proteins were ordered to help yield a diagnosis of malignancy. Lab values were evident for an elevated uric acid, ferritin, and a decreased phosphorus, vitamin D, which are illustrated in Table 1.

**Table 1:** Significant lab results during hospitalization μL: microliter, U: units, g: gram, dL: deciliter, mEq: milliequivalent, mm: millimeters, mmol: millimole, mg: milligram, mL: milliliter

Blood test results	Patient value	Reference range
Calcium (mg/dL)	13	8.5-10.5
Phosphorous (mg/dL)	1.9	2.4-4.4
Vitamin D (ng/mL)	15.1	30.0-100.0
Parathyroid hormone (pg/mL)	9.61	15-65
Parathyroid hormone-related protein (pg/mL)	62	11-20
Uric acid (mg/dL)	11.6	2.6-7.2
Ferritin (ng/mL)	527.7	20-300
C-reactive protein (mg/dL)	10.29	<0.5
Erythrocyte sedimentation rate (mm/Hr)	66	0-20
CA-125 (U/mL)	1020	<35

The patient underwent dilation and curettage (D&C) and hysteroscopy, which showed no resectable lesions with low suspicion for uterine cancer. A Mirena intrauterine device was placed to control the bleeding, which shortly thereafter significantly decreased. At that time, a breast ultrasound and a skin biopsy were ordered.

**Figure 2:**
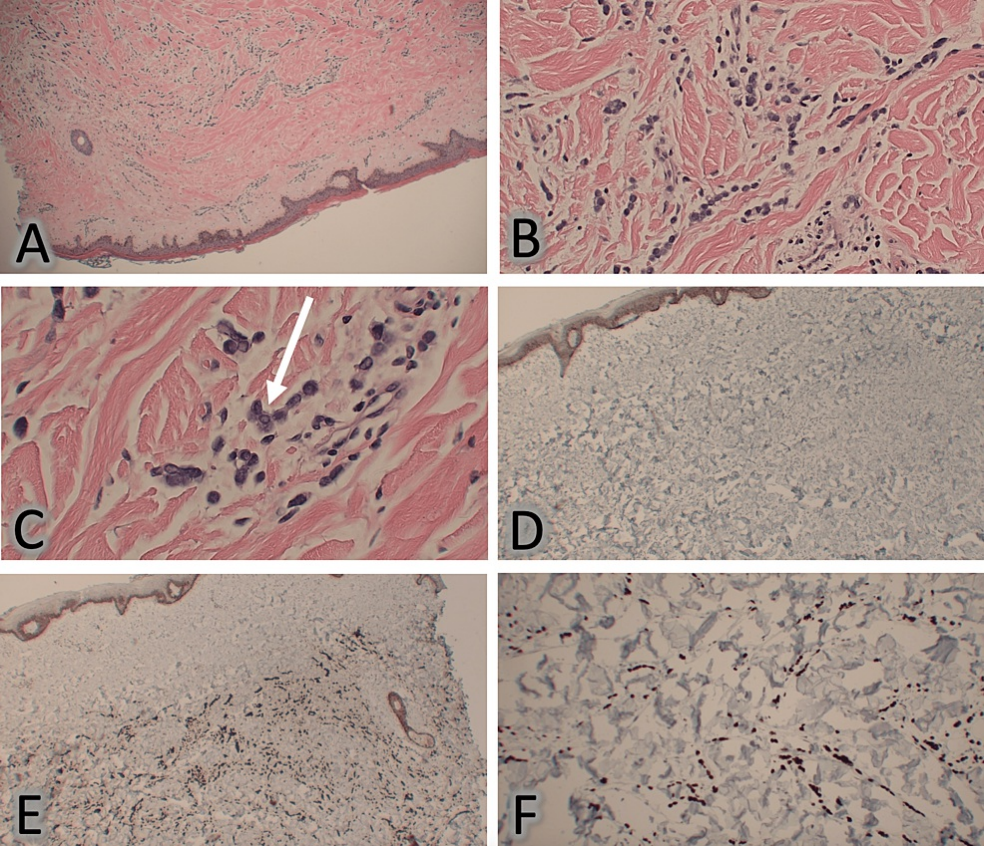
Skin biopsy of diffuse indurated cutaneous nodules showing dermal infiltration (A) Low power field of dermis showing abnormal infiltration of small blue cells. (B) Medium power field of small blue round cells, showing a plasmacytoid appearance, in a single file arrangement. (C) High power field of cells showing dark chromatin around a nuclear membrane (white arrow) with eccentric nuclei. (D) Low power field of E-cadherin immunohistochemical stains highlighting a loss of stain in abnormal cell infiltration, suggesting invasive lobular carcinoma. (E) Low power field of pan-keratin immunohistochemical stains highlighting abnormal cell infiltration. (F) ­High power field of the dermis showing abnormal infiltration of small blue cells, with positive nuclear staining for GATA 3.­

**Figure 3:**
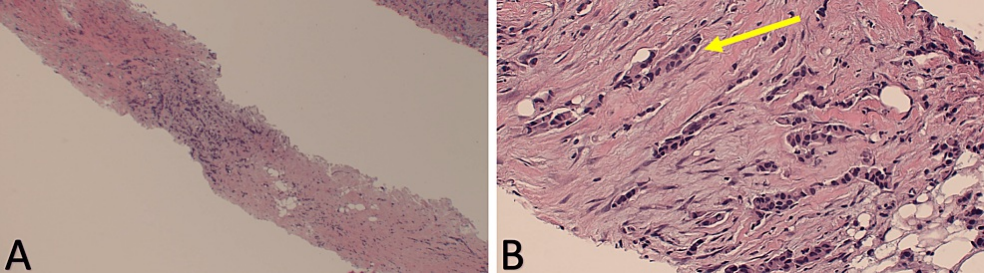
Breast biopsy showing lobular carcinoma of the right breast (A) Low power field histology showing diffuse infiltration of cells. (B) High power field highlighting a single filing arrangement of small blue cells (yellow arrow).

A CT scan of the chest with IV contrast showed a saddle embolus. Breast ultrasound was positive for BI-RAD 3 of the right breast with a small cyst with solid lesions and BI-RAD 3 of the left breast with dilated ducts. However, a skin biopsy was positive for high-grade poorly differentiated infiltrating mammary carcinoma of the lobular type, suggesting metastatic breast cancer, which can be seen in Figure 2. Due to the findings of the skin biopsy, which was concerning for stage IV breast cancer, a breast biopsy (Figure 3) performed revealed estrogen receptor (ER) positive, progesterone receptor (PR) positive, and human epidermal growth factor receptor-2 (HER-2) negative, suggesting invasive mammary carcinoma with mixed ductal and lobular features. An MRI of the brain was negative for brain metastasis. The patient was started on ribociclib and letrozole orally. Due to the inability of the patient to tolerate anything orally, the letrozole was discontinued, and the patient was instead started on fulvestrant. A CT scan of the head and neck was ordered to rule out metastasis to the region and was negative. A gastrostomy tube was ultimately placed for feedings as the patient was unable to open her mouth for oral feeds due to increased tightening around the skin of the mouth.

## Discussion

Melanomas, carcinomas, sarcomas, and hematopoietic malignancies may all cause cutaneous metastatic tumors [4] with the most common being melanomas, squamous cell carcinoma of the head and neck, and breast cancer [1]. Only 2% of cutaneous tumors have cutaneous metastasis, and the incidence of cutaneous metastasis can range from as low as 0.7% to as high as 10% [5]. Cutaneous metastases typically appear two to three years after initial diagnosis and rarely present as the primary manifestation of previously undiagnosed malignancy [2].

Cutaneous metastases initially present most frequently as multiple or single nodules, less frequently as plaques, and least frequently as ulcers [4]. Cutaneous metastases can initially be painless, mobile, and erythematous papules before becoming inflammatory [1]. This early presentation may lead to misdiagnosis as either fibromas, cysts, lipomas, or appendageal tumors [5]. Due to their lymphatic or dermal involvement, cutaneous metastases are often firm and erythematous, which distinguishes them from nonvascular etiologies such as fibroids and cysts [5].

Given our patient’s initial presentation, we considered a differential diagnosis of systemic sclerosis, which includes symptoms such as calcinosis, Raynaud's phenomenon, esophageal dysmotility, sclerodactyly, and telangiectasias [6]. However, the patient was negative for antinuclear antibodies (ANA), anti-Scl-70 antibodies, anti-centromere antibodies, and anti-RNA polymerase III, in addition to a lack of Raynaud’s phenomenon, which aided in ruling out scleroderma and Sjogren's syndrome. The patient had normal kidney function and no osteolytic lesions on the X-ray, which helped in ruling out multiple myeloma. Upon further workup, the patient's presentation was concerning for paraneoplastic syndrome, redirecting the workup from limited systemic sclerosis toward malignancy at the top of the differential. In addition to bilateral breast masses, there were laboratory findings suggestive of hypercalcemia from malignancy. A skin biopsy of diffusely indurated cutaneous nodules showed dermal infiltration by small round neoplastic cells with high nucleus-to-cytoplasmic ratios and hyperchromatic nuclei and nucleoli, suggestive of high-grade, poorly differentiated infiltrating mammary carcinoma of lobular type.

In the United States, invasive breast cancer affects one in eight women in their lifetime [7]. This is significant, as 60% of palpable breast masses in postmenopausal women are malignant, compared to only 10% of palpable breast masses found in women younger than 40 years old [8]. A family history with a first-degree premenopausal relative with breast or ovarian cancer increases the likelihood of genetic mutation; however, our patient did not report a family history of breast cancer. A well-studied association exists with breast cancer gene-1 (BRCA-1) and breast cancer gene-2 (BRCA-2) mutations found in 80-90% of all familial cases of breast cancer. This leads to an increased lifetime risk of breast cancer, often seen at younger ages and found to be more aggressive than sporadic breast cancer [8]. Breast pain is only reported 5% of the time, but locally advanced disease may present with ulceration, fixation to the chest wall, or dimpling of the nipple, known as peau d'orange [7].

Newly diagnosed invasive breast adenocarcinomas require immunohistochemical testing for ER, PR, and immunohistochemical or in situ hybridization (fluorescent or FISH) testing for HER-2 status [8]. Treatments that exist include tamoxifen or aromatase inhibitors for ER- or PR-positive breast cancer and trastuzumab for HER-2-positive breast cancer [8]. Invasive ductal carcinoma is known as the most common type of breast cancer, comprising 40-75% of breast cancer cases, followed by lobular carcinoma (5-15%), mucinous carcinoma (2%), tubular carcinoma (2%), medullary carcinoma (<1%), and metaplastic carcinoma (0.2-5%) [8]. Lobular carcinoma is the second most common breast cancer and features discohesion of monomorphic cells due to inactivation of E-cadherin, a cell adhesion molecule, resulting in a lack of tubular formation. About 60% to 70% of lobular carcinoma tumors are ER-positive and generally HER-2-negative [8].

Our patient’s right breast tumor showed ER and PR positivity and HER-2 negativity. ER-positive and HER-2-negative breast cancers have been found to be associated with activating mutations in PIK3CA, a growth factor receptor signaling molecule [8]. ER-positive and HER-2-negative breast cancers typically progress through the following sequence: flat epithelial atypia, atypical ductal hyperplasia, ductal carcinoma in situ, and finally invasive ductal carcinoma [8]. Given the patient’s ER and PR positivity, the gynecology-oncology team initiated treatment with ribociclib and letrozole. Ribociclib is used for adults with hormone receptor-positive, HER-2-negative breast cancer that is worsening or metastatic and is used in combination with either an aromatase inhibitor or fulvestrant as the endocrine-based therapy [9]. Ribociclib slows the progression of cancer by inhibiting cyclin-dependent kinases 4 and 6, which play a crucial role in signaling pathways leading to cell cycle progression and cellular proliferation. They are activated when binding D cyclins, enabling cancer cells to grow and divide too quickly when overactivated [10]. Letrozole is indicated in hormone-positive breast cancer in post-menopausal women and is given with ribociclib in hormone-positive breast cancer that is HER-2-negative [11]. Letrozole is a third-generation, non-steroidal type 2 aromatase inhibitor that blocks the active site and ultimately the electron transport chain of CYP19A1, preventing the conversion of androgens to estrogens by aromatase. This is mainly responsible for the majority of estrogen production in post-menopausal women. Estrogen-dependent tumors regress with Letrozole therapy, given the resultant reduction in estrogen availability [12].

A note on racial health disparities

The patient in our case is an African American woman. Studies have shown that Black women experience delays in breast cancer diagnostic evaluation and biopsy with total delays of ≥45 days twice as often as White women. When delays in diagnosis reached ≥45 days, a 1.6-fold increase in the odds of breast cancer mortality was identified [13,14]. It is important for us to acknowledge that, in line with the statistics, this patient experienced over a 45-day delay in diagnosis from the onset of her presenting symptom of skin metastasis. Much like the existing literature highlights, this was secondary to barriers to biopsy earlier in her presentation of what was documented as a skin “rash.” Furthermore, it is crucial to recognize that potential bias or racism (implicit or explicit) contributes to healthcare access disparities. In all levels of training, an anti-biased approach to treating patients must be emphasized to optimally provide patients, such as our patient, with the highest quality of care and probability of survival.

## Conclusions

The delayed diagnosis in our patient's case serves as a reminder of the importance of thorough clinical assessment and appropriate referrals, especially when atypical skin manifestations are present. Cutaneous metastases, although relatively uncommon, can manifest in various forms, including nodules, plaques, and ulcers. Early identification and accurate diagnosis are crucial for timely intervention and management. Given the high chance of malignancy, as clinicians, we recommend a skin punch biopsy in patients with skin nodules and a high suspicion of breast cancer at the time of presentation. This can help yield a diagnosis of metastatic malignancy, and ultimately, it will prevent further delays in diagnosis and the initiation of treatment. Overall, this case report underscores the need for heightened clinical vigilance and improved communication within the medical community. By sharing experiences like this, we aim to raise awareness among healthcare professionals about the potential for cutaneous metastases to serve as an early indication of underlying malignancy, urging proactive evaluation and collaboration to enhance patient outcomes.

## References

[1] Lookingbill DP, Spangler N, Sexton FM (1990). Skin involvement as the presenting sign of internal carcinoma. J Am Acad Dermatol.

[2] Schwartz RA (1995). Cutaneous metastatic disease. J Am Acad Dermatol.

[3] Bittencourt Mde J, Carvalho AH, Nascimento BA, Freitas LK, Parijós AM (2015). Cutaneous metastasis of a breast cancer diagnosed 13 years before. An Bras Dermatol.

[4] Reeder-Hayes KE, Mayer SE, Olshan AF (2019). Race and delays in breast cancer treatment across the care continuum in the Carolina Breast Cancer Study. Cancer.

[5] Chernoff KA, Marghoob AA, Lacouture ME, Deng L, Busam KJ, Myskowski PL (2014). Dermoscopic findings in cutaneous metastases. JAMA Dermatol.

[6] Araújo E, Barbosa M, Costa R, Sousa B, Costa V (2020). A first sign not to be missed: cutaneous metastasis from breast cancer. Eur J Case Rep Intern Med.

[7] Adigun R, Goyal A, Hariz A (2022). Systemic sclerosis. StatPearls [Internet].

[8] Alkabban FM, Ferguson T (2022). Breast cancer. StatPearls [Internet].

[9] Scholl AR, Flanagan MB (2020). Educational case: Invasive ductal carcinoma of the breast. Acad Pathol.

[10] (2019). Ribociclib (Kisqali) for the treatment of advanced breast cancer: ribociclib (Kisqali) as part of first-line hormone therapy for advanced breast cancer in women after menopause. InformedHealth.org [Internet].

[11] Yu Q, Sicinska E, Geng Y (2006). Requirement for CDK4 kinase function in breast cancer. Cancer Cell.

[12] Nabholtz JM (2008). Long-term safety of aromatase inhibitors in the treatment of breast cancer. Ther Clin Risk Manag.

[13] Jeong S, Woo MM, Flockhart DA, Desta Z (2009). Inhibition of drug metabolizing cytochrome P450s by the aromatase inhibitor drug letrozole and its major oxidative metabolite 4,4'-methanol-bisbenzonitrile in vitro. Cancer Chemother Pharmacol.

[14] Miller-Kleinhenz JM, Collin LJ, Seidel R, Reddy A, Nash R, Switchenko JM, McCullough LE (2021). Racial disparities in diagnostic delay among women with breast cancer. J Am Coll Radiol.

